# Anticorrosive behavior of a zinc-rich epoxy coating containing sulfonated polyaniline in 3.5% NaCl solution

**DOI:** 10.1039/c8ra00845k

**Published:** 2018-04-10

**Authors:** Feng Yang, Tong Liu, Jingyu Li, Shihui Qiu, Haichao Zhao

**Affiliations:** Shenyang University of Chemistry and Technology, School of Materials Science and Engineering Shenyang 110142 P. R. China; Key Laboratory of Marine Materials and Related Technologies, Zhejiang Key Laboratory of Marine Materials and Protective Technologies, Ningbo Institute of Materials Technology and Engineering, Chinese Academy of Sciences Ningbo 315201 P. R. China zhaohaichao@nimte.ac.cn +86-574-86685159 +86-574-86657094

## Abstract

An epoxy zinc-rich composite coating containing self-doped conducting sulfonated polyaniline (SPANi) nanofiber was prepared and the corrosion resistance of as-prepared coatings on Q235 substrate studied by open circuit potential (OCP), electrochemical impedance spectroscopy (EIS) and scanning vibrating electrode technique (SVET). Results suggested that a zinc-rich coating with addition of 1.0 wt% SPANi could enhance the cathodic protection time and barrier performance. To study corrosion diffusion, artificial scratch and adhesion strength were investigated *via* the salt spray test and pull-off test, respectively. Finally, the passivating action of coatings was demonstrated by analyses of corrosion products *via* X-ray diffraction spectroscopy.

## Abstract 2

An epoxy zinc-rich composite coating containing self-doped conducting sulfonated polyaniline nanofiber was prepared and the corrosion resistances of as-prepared coatings on Q235 substrate was studied by OCP, EIS and SVET.

## Graphical abstract

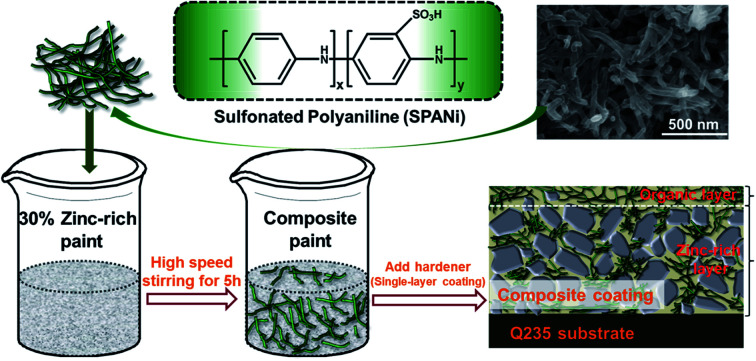

## 1. Introduction

Metal corrosion is a pressing issue facing many industries and can cause economic losses. Epoxy-based organic coatings have been used widely to protect metals in corrosive environments. Overall, organic protective coatings have three functions: barrier, sacrificial and inhibitory action.^1–8^ Most organic coatings can act as a barrier film to prevent the diffusion of corrosive substances such as H_2_O and various ionic solutes to the surface of steel. Moreover, an organic coating can act as a reservoir for corrosion inhibitors or pigments which can slow down the rate of corrosion at coating defects and anodic corrosion sites.^9,10^ For this purpose, many metal- or metal oxide-based pigments have been developed as pigments for epoxy anticorrosive coatings. In terms of zinc fillers, the corrosion resistance performance of zinc in marine environments is superior because the corrosion rate of zinc in a marine atmosphere is usually lower than 1/10 of the corrosion rate of steel.^11^ As a traditional cathodic protective filler with a high utilization rate in industry, active metal zinc powder was regarded as an anode connected with a Fe substrate to form a “corrosion couple”. This leads to a much lower corrosion potential than the self-corrosion potential of Fe, therefore, Fe is protected as a cathode.^12–17^ However, there are also some shortcomings in the zinc-rich coating: on the one hand, the conductivity among zinc particles and between zinc and iron in the coating mainly depends on the contact of a large amount of zinc powder (content of zinc particles in dry film is 70–85%). It produces the higher porosity of coating owing to a large amount of zinc particles and the resulting poor shielding effect of the coating. On the other hand, zinc corrosion products such as ZnO and Zn(OH)_2_ are formed during cathodic protection.^18^ Although it can provide some shielding protection, it leads to the rapid failure of the coating due to the decrease of the connection among the zinc particles.^19–22^ Zinc-rich coatings have been largely limited in application *via* their high porosity, heavy weight and poor environmental protection. Therefore, reducing the amount of zinc powder or increasing the rate of cathodic protection has become an important research direction in the development of new zinc-rich coatings.

Polyaniline (PANi) and PANi derivatives, as a class of conducting polymers, are usually used in corrosion protection.^23,24^ In view of its advantages of simple synthesis, good electrochemical activity and chemical stability, it is generally believed that PANi-based coatings can provide excellent corrosion protection by inhibiting the penetration of corrosion medium, and form passive oxide films on the metal surface.^25,26^ However, pure PANi nanofillers often have problems such as poor solubility and poor interfacial adhesion, which greatly affect the corrosion resistance of coatings. To address this issue, several strategies have been adopted in recent years, including the incorporation of soluble functional groups into the side chain,^27–30^ the selection of appropriate dopants^31–35^ and composite preparation with other well-dispersed nanoparticles.^36–39^

To improve the solubility of PANi and enhance the cathodic protection efficiency of zinc-based organic coatings, we introduced a good-solubility conducting nanofiller into the 30% zinc-rich coating. In detail, sulfonated polyaniline (SPANi) was prepared by introducing sulfonic groups into the main chain of PANi, and the composite coatings were obtained by addition of SPANi with different content in a commercial 30%-zinc-rich coating. The corrosion protection behavior of pure zinc-rich coating, 0.5 wt%, 1.0 wt% and 2.0 wt% nanofiber composite coatings in 3.5 wt% NaCl as a function of immersion time were evaluated by open circuit potential (OCP), electrochemical impedance spectroscopy (EIS), scanning vibrating electrode technique (SVET), salt spray test and pull-off test, respectively. Finally, scanning electron microscopy (SEM) and X-ray diffraction (XRD) spectroscopy were used for investigation of the final corrosion product, and the corrosion mechanism of the coating with SPANi nanofiber was postulated.

## 2. Experimental section

### 2.1 Materials

A commercial anticorrosion paint of industrial steel structure containing 30 wt% zinc dust (HY06-03A) and assorted curing agent (HY06-03B) was purchased from Jiangsu Jinhai Fuqiang Technology (China). Aniline, aniline dimer, ammonium persulfate (APS) and *o*-aminobenzenesulfonic acid (ASA) were provided by Aladdin Industrial Corporation (China). Absolute ethanol and hydrochloric acid were bought from Sinopharm Chemical Reagents. All reagents and chemicals were used without purification. Q235 steel electrodes (working area: 1.0 cm × 1.0 cm) were used as the experimental metal substrate (brand of Q235 steel: Fu Shishipian, China).

### 2.2 Synthesis of SPANi nanofiber

The SPANi nanofiber was obtained through chemical oxidative copolymerization, as reported previously.^30,40^ In detail, 2.5 mmol of ASA and 5.0 mmol of aniline were wholly dissolved in acidic medium (25 mL of 1 N HCl). Then, 0.1875 mmol of aniline dimer was mixed with the obtained solution with continuous magnetic stirring (brand of magnetic stirring apparatus: IKA, China). The mixed solution comprising 5.0 mmol of ammonium persulfate and 25 mL of 1.0 N HCl was promptly added to the monomer solution described above and the final solution was stored at 5 °C for 48 h after vigorous stirring for ≈20 s. The final reactive product (dark-green alcoholic slurry) was collected by a Buchner funnel, washed with 300 mL of deionized water and 500 mL of absolute ethanol, and dried to constant weight.

### 2.3 Preparation of SPANi/zinc-rich coatings

**Fig. 1:**
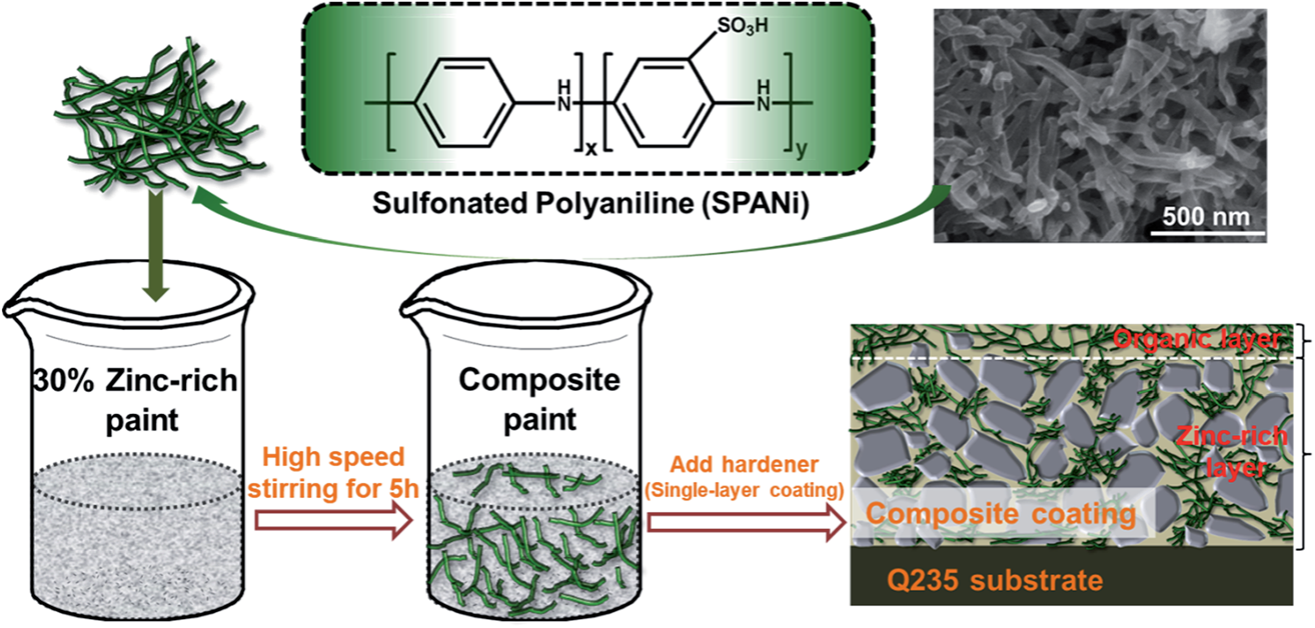
Structural formula and SEM images of water-soluble SPANi nanofiber and coating preparation of zinc-rich/SPANi composite coating.

The contents of SPANi nanofibers were 0.5 wt%, 1.0 wt%, and 2.0 wt% in epoxy zinc-rich paints, and the coating samples were designated as SPANi-0.5, SPANi-1.0 and SPANi-2.0, respectively. By comparison, a pure zinc-rich epoxy coating without SPANi was named “SPANi-0”. First, the weighed SPANi was mixed with 30% zinc-rich epoxy coating by a high-speed agitator (PI7500, China) at 1200 rpm for 5 h (Fig. 1). Then, the hardener (1/10 of weight of zinc-rich epoxy coating) was added and the epoxy diluent was used to adjust viscosity. The composite paint was coated on Q235 steel electrodes by a bar coater (OSPXB, China) and the paint films were completely cured at room temperature for 1 week (actual thickness of the coatings measured was around 40 ± 2 μm).

### 2.4 Characterizations

#### 2.4.1 Characterizations for SPANi nanofiber and composite anticorrosive coatings

Paint diffusion was carried out by a high-speed agitator (PI7500). Coating thickness was obtained *via* a coating thickness instrument (FY2050). SEM images of SPANi nanofibers and the fracture surfaces of different coatings were taken by S-4800 (Hitachi, Japan) and Quanta FEG 250 (FEI, USA), respectively.

#### 2.4.2 Corrosive studies for composite anticorrosive coatings

A three-electrode system (counter electrode: Pt plate; reference electrode: saturated calomel electrode (SCE); working electrode: coating electrodes) was used for measurement of electrochemical data (OCP and EIS) *via* a CHI-660E electrochemical workstation at different immersion times in 3.5 wt% NaCl solution (frequency range for EIS: 10^−2^–10^5^; fitting software: ZsimDemo 3.30).

The corrosive protection behavior of composite coatings was tested by salt spray studies. The salt spray was 3.5 wt% NaCl solution, and the composite paint was coated on Q235 steel sheets with two layers of paint films (thickness of both layers: 150 μm). A neutral salt fog cabinet (CCT1100) was made by Q-Lab Corporation (salt spray test operative standards: ISO 3768-1976, GB 6461-86 and GB 6458-86; China). After 400 h of the salt spray test, an adhesion test was done on the damaged steel sheets by a manual operation adhesion tester (AT-M). SVET was done in a VersaSCAN micro-scanning electrochemical workstation (AMETEK, USA). Along the *Z*-axis direction (scan working area: 2.0 mm × 2.0 mm *vs.* 21 × 21 points *X*-axis and *Y*-axis) and 1.5 mm of artificial scratch was used in 3.5 wt% NaCl solution for this test (SVET parameters: vibration frequency = 80 Hz; amplitude of vibration = 30 μm; scanning rate = 100 μm s^−1^). Test data were analyzed and calculated by the Versa Scan software (VersaSCAN Application).

#### 2.4.3 Characterizations of corrosion products of composite anticorrosive coatings

The corrosion products of composite anticorrosive coatings on the metal surface were characterized by SEM, Raman spectroscopy and XRD spectroscopy. SEM images of the morphology of corrosion products were obtained by Quanta FEG 250 (FEI). The XRD spectroscopy patterns were tested at a scan range of 2*θ* from 20° to 65° *via* a D8 DISCOVER diffractometer (Germany).

## 3. Results and discussion

### 3.1 Characterization of SPANi nanofiber and SPANi/zinc-rich composite coatings

The structural formula of SPANi nanofiber and the coating preparation procedure are shown in Fig. 1. Furthermore, our characterization studies for this typical SPANi have been published.^40,41^ The as-prepared bottle-green SPANi possessed good solubility, electrochemical activity and electrical conductivity. The morphology of the as-prepared SPANi sample according to SEM are exhibited in Fig. 1. It displays a typical nanofibrous structure with an average diameter of 50 nm and length of ≈700 nm. This tiny SPANi nanofiber served as filler for the zinc-rich epoxy coating to improve corrosion resistance.

**Fig. 2:**
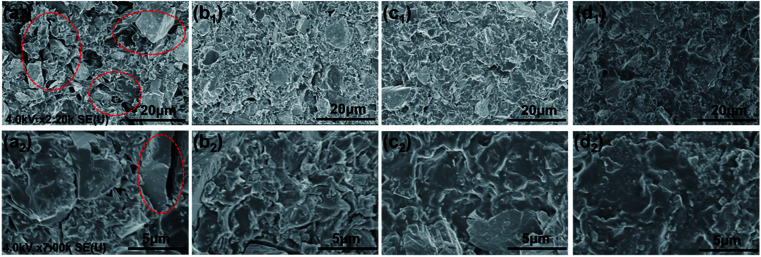
Typical SEM images of fracture surfaces for (a) pure epoxy zinc-rich anticorrosion coating; (b) 0.5 wt% SPANi; (c) 1.0 wt% SPANi; (d) 2.0 wt% SPANi zinc-rich coatings.

The morphology of the fracture surfaces of different coatings was obtained by SEM to study the dispersion ability of SPANi nanofibers within the composite coatings (Fig. 2). With respect to the cross-section of a pure zinc-rich anticorrosive coating, the granulated zinc powder was easily observed in the coatings (a_1_, a_2_), which resulted in many ineluctable holes and cracks among the zinc powder (red circles). These inevitable defects can impact on the cathodic protection of zinc powder and the coating performance. For samples containing SPANi nanofibers (b, c and d), these defects could be filled by an appropriate amount of conducting nanofiller. In terms of dispersibility, finely dispersed coatings with decreased porosity 1.0 and 2.0 wt% nanofibers could be obtained (Fig. 2c and d).

### 3.2 OCP measurement

**Fig. 3:**
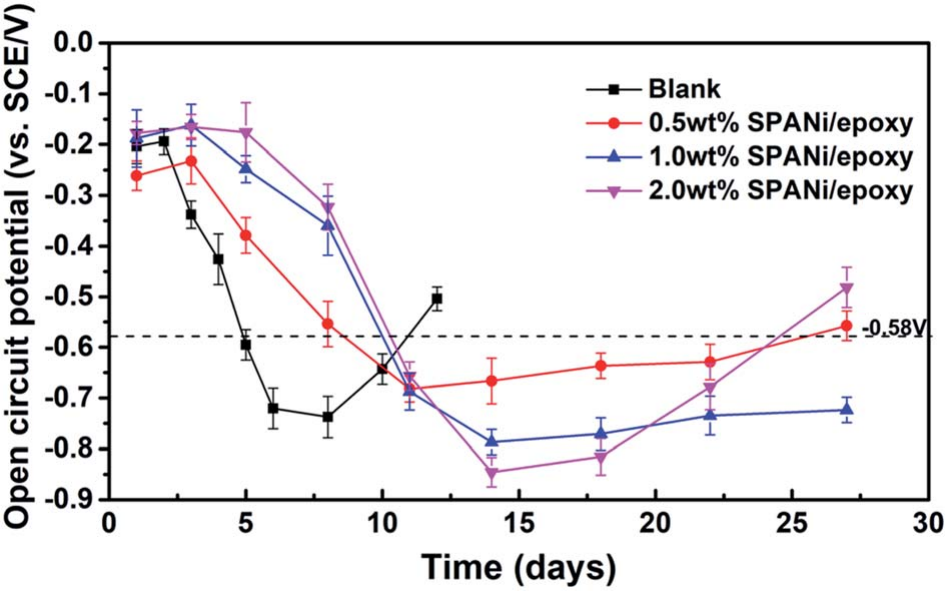
Open circuit potential for the pure zinc-rich coating, 0.5 wt%, 1.0 wt% and 2.0 wt% SPANi zinc-rich coatings after different immersion times in 3.5 wt% NaCl solution.

OCP variation as a function of the immersion time for composite coatings in 3.5 wt% NaCl solution implied the anti-corrosive performances of the organic layer and cathodic protection area (zinc-rich layer). As shown in Fig. 3, in the initial immersion stage, the OCP values of the pure epoxy zinc-rich anticorrosion coating and the other three SPANi composite coatings showed a decrease with permeation of an aggressive medium. With an increase in immersion time, the OCP of all of coatings was below that of iron (OCP = −0.580 V), revealing that the corrosion solution had reached a zinc-rich layer.^14^ In terms of Q235 steel with a pure zinc coating, at the beginning of corrosion, the electrolyte seeped slowly into the organic layer, and the cathodic protection stage occurred at ≈6 days (about −0.720 V) because the porosity of the zinc-rich coating led to acceleration of the electrolyte diffusion rate.^6^ During the immersion process for cathodic protection, the OCP values tended to increase, which was attributed to the consumption of zinc particles. In detail, partial zinc powder as the positive pole was consumed to form a series of oxidation products of zinc. It could reduce the connection between the zinc powder and the formed zinc oxide layer to cause a positive shift in the OCP. After 12 days of immersion, the OCP value of the blank coating was up to −0.504 V, and some bubbles on the surface of the coating were visible. By comparison, the three composite coating samples (SPANi-0.5, SPANi-1.0 and SPANi-2.0 coatings) showed better corrosion protection at ≈27 days of immersion. In particular, the SPANi-1.0 coating exhibited the slowest increase in OCP at 27 days of immersion, implying that zinc powder could enhance the corrosion efficiency significantly upon addition of a moderately conducting polymer.^14,42^

### 3.3 EIS studies

**Fig. 4:**
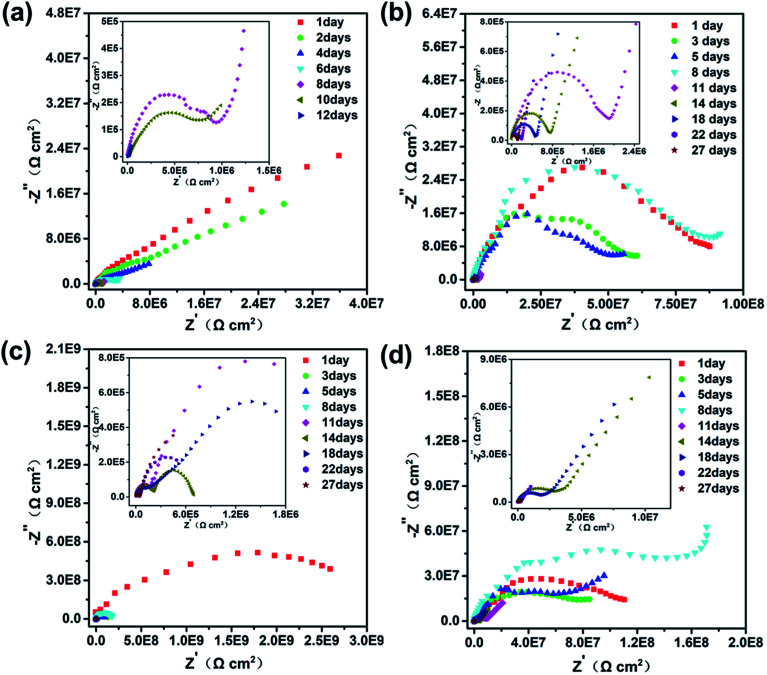
Nyquist plots of (a) pure zinc-rich coating, (b) 0.5 wt%, (c) 1.0 wt% and (d) 2.0 wt% SPANi zinc-rich coatings during 27 days of immersion in 3.5 wt% NaCl solution.

**Fig. 5:**
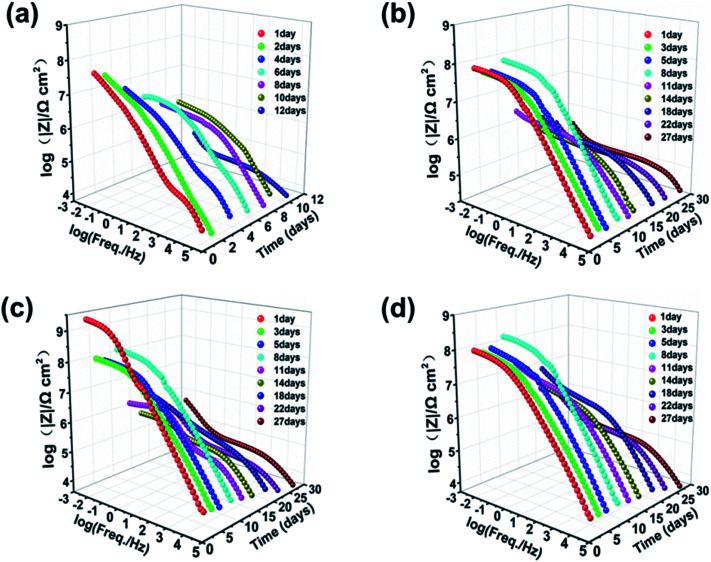
Bode plots of (a) pure zinc-rich coating, (b) 0.5 wt%, (c) 1.0 wt% and (d) 2.0 wt% SPANi zinc-rich coatings during 27 days of immersion in 3.5 wt% NaCl solution.

EIS data were used to represent the corrosion protection behaviors of Q235 steel electrodes coated with neat zinc-rich coating, 0.5 wt%, 1.0 wt% and 2.0 wt% SPANi zinc-rich coatings. The Nyquist and impedance-Bode plots of the different coating samples as a function of immersion time in 3.5 wt% NaCl are exhibited in Fig. 4 and 5 (the duration of immersion of the neat zinc-rich epoxy coating and composite coatings were 12 days and 27 days, respectively). In terms of the neat zinc-rich corrosion coating (Fig. 4a), the Nyquist plot displayed shrinking capacitive arc radii during the entire immersion, implying a decline of anticorrosion capability for a neat zinc-rich epoxy coating.^43^ As seen in Fig. 4a, the capacitive arc radii reduced rapidly at 12 days of immersion. This was because the zinc-rich corrosion coating had a porous structure that led to a decrease in contact between zinc particles, which eventually caused obvious weakening of cathodic protection. For three composite coatings, the capacitive loop radii had a significant increase after 8–10 days of immersion, suggesting that the aggressive medium just infiltrated the zinc layers, and then the oxide (*e.g.* ZnO, Zn(OH)_2_) was formed to fill the subtle defects in the zinc-rich layer. Particularly for 0.5 and 1.0 wt% samples, the radii of Nyquist plots at day 8 expanded beyond that of day 1 as a result of the passivation product formed by zinc oxide/hydroxide and interlayer SPANi. For the Bode plots in Fig. 5, a higher impedance value at a low measured frequency (|*Z*|_f=0.01 Hz_) indicated better coating shielding.^44–46^

The Bode-impedance value of the pure zinc-rich coating decreased gradually from 3.59 × 10^7^ (day 1) to 3.83 × 10^4^ Ω cm^2^ (day 12), implying that the SPANi-0 coating had poor anticorrosion performance in the corrosion medium due to the porosity of the coating and weak cathodic protection (Fig. 5a). By contrast, for initial immersion, the values of |*Z*|_f=0.01 Hz_ for SPANi-1.0 and SPANi-2.0 were up to 2.59 × 10^9^ Ω cm^2^ and 1.11 × 10^8^ Ω cm^2^, respectively, which were higher than those of the neat zinc-rich sample and SPANi-0.5 (8.76 × 10^6^ Ω cm^2^) by one or two orders of magnitude. The impedance plot of SPANi-1.0 (Fig. 5c) showed an obvious decline in |*Z*|_f=0.01 Hz_ from 1 day (2.59 × 10^9^ Ω cm^2^) to 5 days (9.87 × 10^7^ Ω cm^2^). This result demonstrated that an appropriate amount of nanofibers could enhance the corrosion resistance of the organic layer to some extent.^40^ In addition, after around 8 days of immersion, there was a different degree of increase for |*Z*|_f=0.01 Hz_ of the three composite coatings. This was due to filling of a Zn-containing oxide to make the coating have higher protection performance, and showed that the anode reaction (Zn_anode_ − Fe_cathode_) had begun. In the following immersion process (8–27 days), the gradually decline in impedance denoted reduction of reactive zinc particles in cathodic protection. After 27 days of immersion, the final |*Z*|_f=0.01 Hz_ values of SPANi-0.5, SPANi-1.0 and SPANi-2.0 were 2.97 × 10^5^, 4.84 × 10^5^ and 3.08 × 10^5^ Ω cm^2^, respectively, which are typical for epoxy zinc-rich coatings.^7,47–49^ In summary, Q235 steel coated with 1.0 wt% SPANi possessed a better impedance value and cathodic protection ability, which manifested as the best corrosion protection performance.

**Fig. 6:**
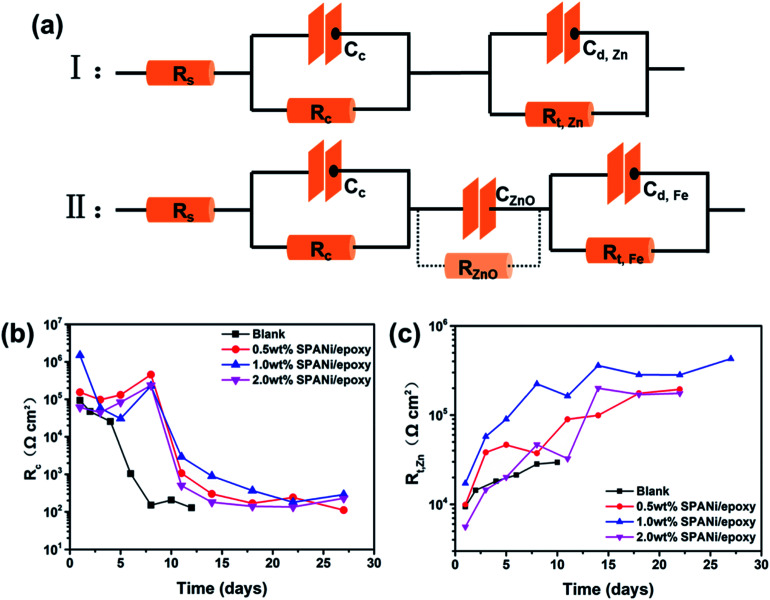
(a) Equivalent electric circuits (I, II) of the coatings and fitting results (b and c) of the collected EIS results (*R*_c_, *R*_t,Zn_) of different coatings as a function of immersion time.

Fig. 6a (I, II) show the EIS-fitted equivalent electric circuits (EECs) used to obtain a series of electrochemical corrosion parameters by ZsimDemo 3.30 software. In the equivalent circuits of Fig. 6a, the *R*_s_, *R*_c_, *C*_c_, *R*_t,Zn_, *C*_d,Zn_, *R*_ZnO_, *C*_ZnO_, *R*_t,Fe_ and *C*_d,Fe_ represent the solution resistance, organic layer resistance, organic layer capacitance, zinc powder charge transfer resistance, corrosion reaction charge transfer resistance (Zn), double-layer capacitance of the zinc powder surface, zinc oxide resistance, zinc oxide capacitance, corrosion reaction resistance (Q235 substrate surface) and double-layer capacitance (Q235 substrate surface), respectively.^50^

From the perspective of coating structure, the organic layer was relatively thinner than the zinc-rich layer, so the electrolyte could reach the surface of zinc particles rapidly to react.^19,49,51^ This effect resulted in the emergence of two time constants at the initial immersion in Fig. 6a (I). Overall, circuit I was a series circuit with two time constants and showed that the order of permeation was divided into two layers. As seen in Fig. 6b, the organic layer resistance (*R*_c_) declined quickly until the change was stopped at 8 days, indicating that the water had completely infiltrated into the organic coating. The *R*_c_ value of composition coatings had the same variation trend as that of the pure zinc-rich coating. However, unlike the blank, the organic layer resistance improved in the first 8 days because SPANi nanofibers enhanced the compactness of the organic layer and induced the generation of zinc oxide. For the late stage of the coating immersion (circuit II), the zinc particles reacted completely and zinc oxide never participated in other electrochemical reactions, so the zinc oxide resistance (*R*_ZnO_) was regarded to be infinity and ignored. At this time, the appearance of *R*_t,Fe_ and *C*_d,Fe_ demonstrated that the aggressive medium had reached the steel-substrate surface, illustrating that the coating had lost its protection. In addition, the corrosion reaction charge transfer resistance of zinc powder (*R*_t,Zn_) reflected the effective protection time of zinc powder for different coatings. As seen in Fig. 6c, the highest *R*_t,Zn_ value and the longest cathodic protection time were observed for the 1.0 wt% SPANi nanofiber zinc-rich coating.

### 3.4 Salt spray test and pull-off test (after salt spray)

**Fig. 7:**
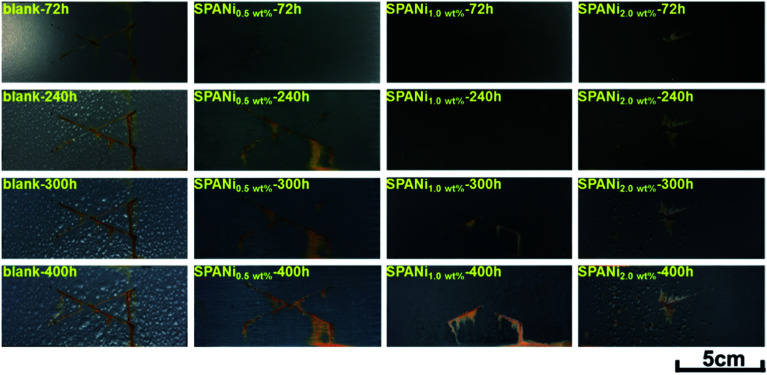
Typical optical images of blank, 0.5 wt%, 1.0 wt% and 2.0 wt% of SPANi zinc-rich coating samples after 72, 240, 300 and 400 h of salt spray tests (test length of X-scribes: 5 cm).

To further investigate the corrosion protection performance of composite coatings, the visual performances of a salt spray study (X-scribes method) at exposure times of 72, 240, 300, and 400 h in 3.5 wt% NaCl salt spray were conducted, as shown in Fig. 7 and Table 1. The duration of cathodic protection of the zinc-rich epoxy coating was affected significantly by the addition of SPANi nanofibers. For the pure zinc-rich epoxy sample, light-red rust appeared around X-scribes after 72 h of salt spray exposure and several coating blisters were observed on the surface of steel sheets after 240 h, suggesting that the interior coating had degraded and no longer provided protection for the steel substrate. With increasing time, a vast area of blisters and red rust appeared after 400 h, suggesting that the Q235 substrate was very corroded. In terms of the SPANi coating sample, the 0.5 wt% SPANi coating had obvious red rust on the X-scribes after 240 h of exposure, but there was no blistering on the surface of the composite coating after 400 h, illustrating that the composite coating could inhibit diffusion of the corrosion medium. However, for the SPANi-2.0 sample, a small range of corrosion diffusion and blistering were observed after 300 h of exposure due to generation of coating defects with excess SPANi. Compared with other coatings, the 1.0 wt% SPANi zinc-rich sample had notably improved salt fog resistance property with less rust and without blisters, which demonstrated the positive role of SPANi nanofiber on cathodic protection. In conclusion, a zinc-rich coating containing 1.0 wt% SPANi nanofiber can enhance the sacrificial efficiency of zinc powder remarkably.

**Table 1:** Results of the salt spray test

Coating sample	Parameters of corrosion performance	
	Blistering circumstances (400 h)	Corrosion behavior at scribes
SPANi-0	Many blisters	Obvious red rust after 240 h
SPANi-0.5	Without blister	Obvious red rust after 240 h
SPANi-1.0	Without blister	Lesser red rust after 300 h
SPANi-2.0	Some blisters	Rust diffusion after 240 h

**Fig. 8:**
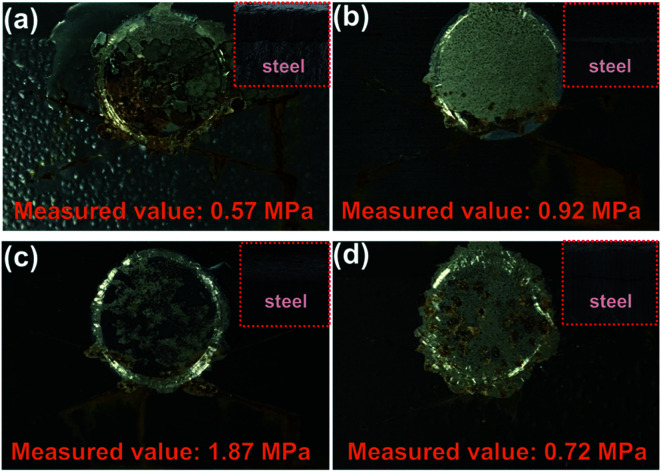
Results of the pull-off test after 400 h of the salt spray test for blank, 0.5 wt%, 1.0 wt% and 2.0 wt% of SPANi zinc-rich coating samples (the measured value is the average value obtained from three pull-off tests in three parallel samples).

The pull-off test was used to obtain the adhesion force (unit: MPa) of different coatings after 400 h of exposure to salt spray. For the measurement results shown in Fig. 8, the blank had the lowest adhesion strength (measured value: 0.57 MPa), compared with those of SPANi-0.5 (0.92 Mpa), SPANi-1.0 (1.87 MPa) and SPANi-2.0 (0.72 MPa), respectively, thereby revealing different degrees of improvement. In terms of corrosion diffusion, as seen in Fig. 8a (SPANi-0) and Fig. 8d (SPANi-2.0), a corrosion area was observed clearly at the circle region of the pull-off test, resulting in a decrease in the coating adhesion strength. The SPANi-1.0 sample, which possessed the highest adhesion strength, did not show corrosion spread and produced a small corrosion product at X-scribes. Consequently, in accordance with the improvement in cathodic protection efficiency, the corrosive spread at the artificial scratch was slowed down *via* addition of 1.0 wt% SPANi nanofibers in a zinc-rich coating.

### 3.5 SVET studies

**Fig. 9:**
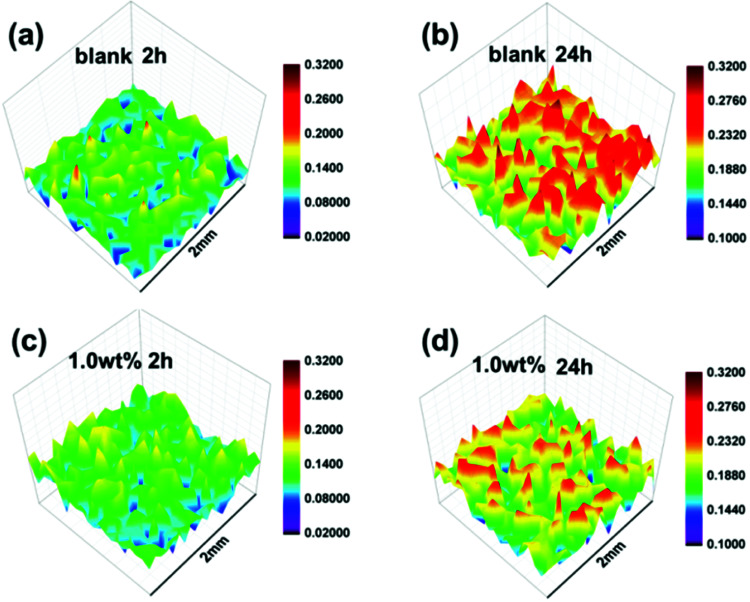
SVET 3D current density distribution maps of coated steel immersed in 3.5 wt% NaCl solution for 2 h and 24 h. Blank sample: (a), 2 h and (b), 24 h; 1.0 wt% of SPANi zinc-rich coating sample: (c), 2 h and (d), 24 h; unit of current density = μA cm^−2^.

As shown in Fig. 9, SVET was used to convert the change in the corrosive potential signals around the artificial scratch (scratch length: 1.5 mm) into current density signals *via* exposure to 3.5% NaCl solution. With regard to the blank coating (Fig. 9a), an anodic current density peak map was obtained after 2 h of immersion (variation of anodic current density: 0.02–0.18 μA cm^−2^). This was similar to the initial region of anodic current density of SPANi-1.0 (Fig. 9c), suggesting that inchoate anodic dissolution occurred upon an artificial scratch. With increasing immersion time (2–24 h), an obvious variation in corrosion current density (0.10–0.30 μA cm^−2^) and noticeable corrosive peak map were observed in Fig. 9b, revealing the aggravation of corrosion for the metal substrate. Compared with SPANi-0, the coating containing 1.0 wt% nanofiber possessed a lower anodic current density (0.10–0.24 μA cm^−2^) and a smaller corrosion variation was observed around the scratch (Fig. 9d), implying that the anodic reaction activity was restrained at the artificial scratch during 24 h of immersion time. This result showed that the conducting nanofiber added to the zinc-rich coating had helped to retard corrosion of coating defects.

### 3.6 Studies on corrosion products

**Fig. 10:**
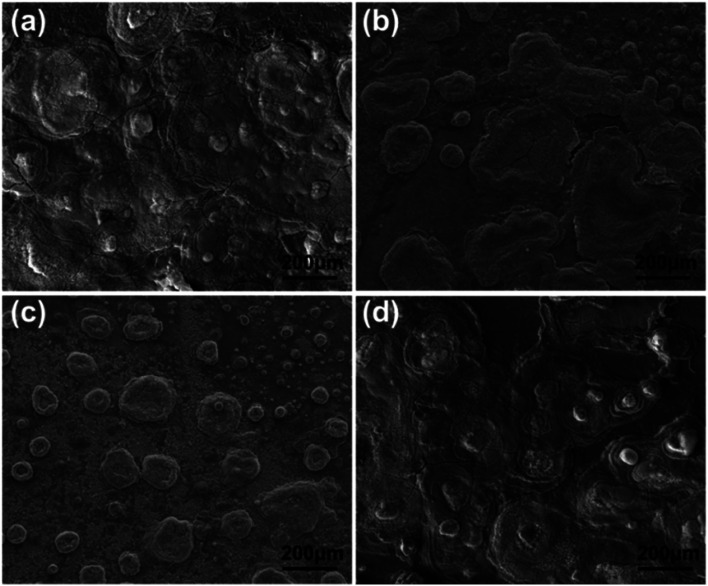
SEM images of corrosion products on a steel substrate coated by blank (a), 0.5 wt% (b), 1.0 wt% (c) and 2.0 wt% (d) of SPANi zinc-rich coating samples after 27 days of immersion.

**Fig. 11:**
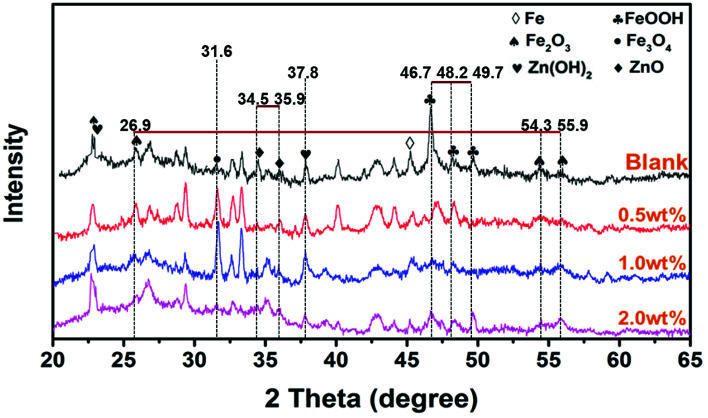
XRD spectroscopy patterns of corrosion products on a steel substrate of pure epoxy, 0.5, 1.0 and 2.0 wt% SPANi zinc-rich coatings after 27 days of exposure to 3.5% NaCl solution.

To further investigate the corrosive process on steel, the morphology images of rust products beneath neat epoxy zinc-rich and nanofiber coatings (SPANi-0.5, SPANi-1.0 and SPANi-2.0) are shown in Fig. 10a–d, respectively. Compared with the large area of corrosion of the blank, SPANi-0.5 and SPANi-2.0 samples, a small quantity of a “round-cake-shape” corrosion product was observed only on the substrate surface of the 1.0 wt% nanofiber epoxy zinc-rich coating, indicating that the SPANi-1.0 coating had the weakest degree of corrosion in the different samples. In addition, the XRD spectroscopy patterns of the corrosion products of different composite coatings are exhibited in Fig. 11. They displayed the diffraction peaks associated with FeOOH, Fe_2_O_3_, Fe_3_O_4_, Fe_2_(OH)_3_Cl, Zn(OH)_2_ and ZnO after 27 days of immersion. As important final products of corrosion, ZnO, Fe_2_O_3_ and Fe_3_O_4_ are the key metal oxides for formation of a passivation film.^7,52^ In addition, FeOOH and Zn(OH)_2_, as the intermediate products of corrosion, are also important indices to evaluate the corrosion degree of metals. Compared with the neat anticorrosion zinc-rich coating, the diffraction peaks related to ZnO (2*θ* ≈ 34.5°and 35.9°) phases decreased slightly and the peak intensity of Zn(OH)_2_ (2*θ* ≈ 37.8°) was approximately equal, revealing that moderate nanofibers could improve the cathodic protection efficiency of composite coatings. Moreover, the intensities of the peaks (2*θ* ≈ 46.7°, 48.2° and 49.7°) associated with FeOOH phases declined significantly with the addition of SPANi nanofiber. However, in terms of composite coatings, the intensity of diffraction peaks related to the Fe_3_O_4_ (2*θ* ≈ 31.6°) phase, especially SPANi-1.0, had an obviously higher intensity compared with that of the pure zinc coating. This result indicated that the reactivity of the intermediate product (FeOOH phase) was promoted after addition of SPANi nanofiber due to its excellent redox properties, which contributed to the formation of passivation films. These results showed that the addition of 1.0 wt% SPANi to the coatings could promote formation of a passivation layer and enhance the corrosion efficiency of the zinc-rich coating.

### 3.7 Protective mechanism for composite coatings

**Fig. 12:**
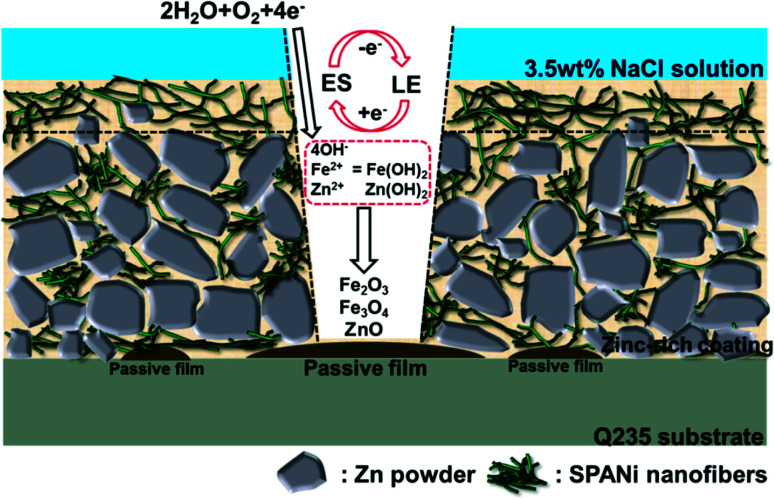
Proposed mechanism of corrosion of SPANi zinc-rich coating in 3.5 wt% NaCl solution (one layer of composite paint).

A mechanism of corrosion for a SPANi zinc-rich coating is shown in Fig. 12. For the initial stage of corrosion, the nanofiber in the organic layer of the surface acted as a barrier and prevented seepage of the corrosion medium.^25,53^ In addition, the excellent redox property of SPANi nanofiber had a protective effect on the metal substrate by generating a passive film. Specifically, the cathodic reaction of SPANi also occurred. This led to the transfer of the emeraldine salt (ES; oxidation state) form of SPANi to the reduced form of leucoemeraldine (LE; reduction state) by the gaining of dissociative electrons from the anodic dissolution of zinc powder in the coating and iron substrate. The redox activity of SPANi led to an isolated anode/cathode reaction to maintain a supply of OH^−^ (which was necessary for the anodic reaction) and, thanks to auto-oxidation of SPANi nanofiber, the reduction state (LE) of SPANi could return to the oxidation state (ES).^52^ Moreover, with ceaseless increasing of Fe^2+^, Fe^3+^and Zn^2+^ and the push of the redox reaction, increasing amounts of Fe_2_O_3_, Fe_3_O_4_ and ZnO deposited on the metal surface to form oxide passive films (Fig. 10c). For the stage of cathodic protection (shown graphically in Fig. 12), SPANi, as a conducting polymer, acted as a “bridge” and filler to zinc particles, decreasing the coating porosity and enhancing the cathodic protection efficiency because more zinc powder was involved in cathodic protection and because it could reduce the damage wrought by free zinc to the coating.

## 4. Conclusions

A highly soluble and highly dispersible conducting polymer, SPANi, was synthesized and applied as nanofiller for zinc-based epoxy coatings. The corrosion protection of the composite coatings without and with 0.5, 1.0 and 2.0 wt% SPANi nanofiber were studied in 3.5 wt% NaCl solution by OCP, EIS, salt spray test, pull-off test and SVET, respectively. The zinc coating containing 1.0 wt% SPANi nanofiber possessed the best anti-corrosion properties, good protection of scratch corrosion spread performance, and high adhesion strength (after salt spray corrosion). Furthermore, studies of the corrosion products on a steel substrate implied that the passive films (main substance: Fe_3_O_4_; others: Fe_2_O_3_, ZnO) formed on metal surfaces owing to the excellent reversible redox performance of SPANi. Nanofiber addition could evidently enhance effective utilization of zinc powder and decrease the coating porosity, which made SPANi nanofiber a novel conductive filler for enhancement of zinc-based anticorrosive coatings.

## Conflicts of interest

There are no conflicts to declare.

## Supplementary Material
